# Tempol Alters Urinary Extracellular Vesicle Lipid Content and Release While Reducing Blood Pressure during the Development of Salt-Sensitive Hypertension

**DOI:** 10.3390/biom11121804

**Published:** 2021-12-01

**Authors:** Kevin M. Chacko, Mohammad-Zaman Nouri, Whitney C. Schramm, Zeeshan Malik, Lauren P. Liu, Nancy D. Denslow, Abdel A. Alli

**Affiliations:** 1 Department of Physiology and Functional Genomics, University of Florida, Gainesville, FL 32610, USA; kchacko@ufl.edu (K.M.C.); whitneyschramm11829@gmail.com (W.C.S.); zmalik456@gmail.com (Z.M.); lpliu13@ufl.edu (L.P.L.); 2 Department of Physiological Sciences and Center for Environmental and Human Toxicology, University of Florida, Gainesville, FL 32610, USA; zaman@phenomixsciences.com (M.-Z.N.); ndenslow@ufl.edu (N.D.D.); 3 Department of Medicine, Division of Nephrology, Hypertension, and Renal Transplantation, University of Florida, Gainesville, FL 32610, USA

**Keywords:** Tempol, ROS, ENaC, MARCKS, salt-sensitive hypertension, extracellular vesicles, lipid

## Abstract

Salt-sensitive hypertension resulting from an increase in blood pressure after high dietary salt intake is associated with an increase in the production of reactive oxygen species (ROS). ROS are known to increase the activity of the epithelial sodium channel (ENaC), and therefore, they have an indirect effect on sodium retention and increasing blood pressure. Extracellular vesicles (EVs) carry various molecules including proteins, microRNAs, and lipids and play a role in intercellular communication and intracellular signaling in health and disease. We investigated changes in EV lipids, urinary electrolytes, osmolality, blood pressure, and expression of renal ENaC and its adaptor protein, MARCKS/MARCKS Like Protein 1 (MLP1) after administration of the antioxidant Tempol in salt-sensitive hypertensive 129Sv mice. Our results show Tempol infusion reduces systolic blood pressure and protein expression of the alpha subunit of ENaC and MARCKS in the kidney cortex of hypertensive 129Sv mice. Our lipidomic data show an enrichment of diacylglycerols and monoacylglycerols and reduction in ceramides, dihydroceramides, and triacylglycerols in urinary EVs from these mice after Tempol treatment. These data will provide insight into our understanding of mechanisms involving strategies aimed to inhibit ROS to alleviate salt-sensitive hypertension.

## 1. Introduction

EVs play an important role in cellular communication [1], differentiation [2], and intracellular signaling [3]. The packaged cargo within these nano-sized vesicles includes nucleic acids, proteins, and lipids [4,5]. EVs are release into the tubular lumen by all cell types expressed within the nephron. Therefore, EVs excreted into the urine contain a mixed population of vesicles with unique cargo. Studies have shown that urinary EVs contain membrane proteins expressed in each segment of the nephron including the epithelial sodium channel (ENaC) expressed in the distal tubule, connecting tubule, and collecting duct.

ENaC plays a vital role in the regulation of total body sodium reabsorption, fluid homeostasis, and blood pressure control [6]. The role of ENaC in the development of salt-sensitive hypertension is generally established [7]. ENaC is positively regulated by the anionic phospholipid phosphates and the adaptor protein MARCKS [8,9].

Nitroxides are compounds that can be converted to hydroxylamines or oxoammonium, which can directly scavenge free radicals [10]. One of the most studied nitroxides is Tempol (4-Hydroxy-2,2,6,6-tetramethylpiperidine-*N*-oxyl), a superoxide dismutase mimetic that can pass directly through biological membranes due to its low molecular weight [11,12]. Tempol is a potent antioxidant, with beneficial biological effects on the heart, kidney, and central nervous system as well as protective effects against radiation [11,12]. Tempol has been shown to protect the kidney from chronic ischemic renal injury and prevents renal dysfunction [13]. Tempol has also been shown to decrease blood pressure in various animal models of hypertension [10,14]. To our knowledge, the effect of Tempol treatment on urinary EV release and composition has not been investigated.

In this study we tested our hypothesis that Tempol treatment alters the lipid profile of EVs excreted unto the urine and we further investigated correlative physiological responses of tempol including reductions in blood pressure, protein expression of ENaC, and its adaptor protein MARCKS. Our data suggest that the packaged cargo within urinary EVs may serve as useful biomarkers to better understand the mechanism of anti-hypertensive drugs using a mouse model.

## 2. Materials and Methods

### 2.1. Animals

129Sv mice were purchased from The Jackson Laboratory (Bar Harbor, ME, USA). Both adult male and female mice 13 months of age were used for various studies under an approved University of Florida’s Institutional Animal Care and Use Committees protocol.

### 2.2. Osmotic Minipump Infusion of Tempol

129Sv mice were given Tempol through osmotic mini pumps (Alzet model 2002, ALZET Osmotic 103 Pumps, Cupertino, CA, USA) according to the manufacturer’s instructions. Briefly, the minipumps were filled with Tempol prepared in DMSO. For one cohort, 2 male and 3 female 129Sv mice of approximately 20–30 g in weight were anesthetized with isoflurane for 5 min before subcutaneous implantation of osmotic minipumps containing Tempol while another cohort of the same number of male and female mice was subcutaneously implanted with minipumps containing 50% DMSO in sterile saline and the mice were maintained in metabolic cages. Each mouse’s surgical incision was closed with 2 sutures. All mice were maintained in metabolic cages following the procedure. The infusion rate was scheduled at 0.5 μL/h for 7 days before the mice were euthanized and the kidneys were harvested for protein. All animals were euthanized between 6 and 9 p.m. by cervical dislocation.

### 2.3. Blood Pressure Measurements

Systolic blood pressure in 129Sv mice was recorded weekly between 6 and 9 p.m. using a mouse tail cuff IITC MRBP System (Life Science Inc., Woodland Hills, CA, USA). The mice were trained to the blood pressure system at the beginning of the study in order to minimize the amount of time needed for subsequent blood pressure recordings.

### 2.4. Metabolic Cage Studies and Animal Diet

129Sv mice were kept on a 12 h light/12 h dark cycle and individually placed in metabolic cages (Ancare; Bellmore, NY, USA) for urine collections for a period of 8 weeks. Mice were given water ad libitum and the mice were initially given a normal salt diet (0.49% NaCl) for 3 weeks followed by a high salt diet (8% NaCl) to promote the development of hypertension. The mice were maintained on a high salt diet for 5 weeks including the last 9 days of the study in which they were subject to Tempol infusion. The AIN-93G Diet (TD.94045.PWD) (Envigo; Madison, WI, USA), agar, and an appropriate amount of NaCl was mixed and heated in diH20 before being dispensed into food cups. Urine excretion and water uptake were recorded daily.

### 2.5. Kidney Homogenate Preparation

Fifty milligrams of kidney cortex snap frozen tissue from 129Sv mice were homogenized in ice-cold Tissue Protein Extraction Reagent (TPER) (Thermo Fisher Scientific, Waltham, MA, USA) containing protease and phosphatase inhibitors. The homogenates were centrifuged at 5000× *g* for 15 min to remove debris before subjecting the supernatants to ultracentrifugation for 30 min at 110,000 g. The resulting pellets were reconstituted in 250 μL TPER.

### 2.6. BCA Assay

BSA standards and a BCA reagent assay kit (Thermo Fisher Scientific, Waltham, MA, USA) were used to determine the concentration of proteins in various samples while following the manufacturer’s instructions with the following modifications. A total of 9 BSA standards were prepared from serial dilutions of a stock 2 mg/mL solution of BSA (Sigma-Aldrich, St. Louis, MO, USA) in reagent grade water. Twenty-five microliters of a 1:10 dilution of each tissue lysate sample and each of the 9 BSA standards were added to a 96 well plate before the addition of 200 μL of BCA assay reagents (Thermo Fisher Scientific, Waltham, MA, USA). A linear regression curve from the optical density readings at 570 nm for the standards was plotted in order to calculate the concentration of each sample.

### 2.7. SDS PAGE, Immunoblotting, and Densitometry

A Criterion system (BioRad, Hercules, CA, USA) was used to resolve 50 μg total proteins on 4–20% Tris Glycine gels (Thermo Fisher Scientific). The proteins were then electrophoretically transferred to a nitrocellulose membrane (Thermo Fisher Scientific) using Towbin buffer (25 mM Tris, 192 mM glycine, 20% (*v*/*v*) methanol) and a Criterion transfer apparatus (BioRad). The membranes were blocked with 5% non-fat dry milk prepared in 1XTBS (BioRad) and then incubated with primary antibody (Flotillin-2 (3244; Cell Signaling), TSG101(ab30871; abcam), annexin-A2 (8235 Cell Signaling), or ENaC α antibody [8] at a 1:1000 dilution in 5% BSA solution prepared in 1X TBS (BioRad) or MARCKS/MLP1 antibody (ab72459; abcam) at a 1:2000 or beta actin-peroxidase antibody (A3854; Sigma) at a 1:25,000 dilution in the same buffer. The membranes were then incubated with a 1:3000 dilution of goat-anti rabbit secondary antibody (BioRad). Finally, each blot was incubated with SuperSignal Pico chemiluminescence solution (Thermo Fisher Scientific) according to the manufactures instructions and then imaged using a ChemiDoc XRS imaging system (BioRad). ImageJ (Version 1.51a, NIH) was used to determine the relative densities of the immunoreactive bands in the Western blots.

### 2.8. Electrolyte Measurements

Urinary sodium and potassium electrolyte concentrations were measured daily using a SmartLyte Electrolyte ISE Analyzer (Diamond Diagnostics, Holliston, MA, USA). Samples were centrifuged at 13,000 rpm for 10 min, diluted in urine diluent (Diamond Diagnostics), and mixed by vortexing for 3 s before being measured.

### 2.9. Urinary EV Isolation

For each phase of the study (before Tempol and after Tempol), EVs were isolated from a total of 10 mL’s of pooled urine collected at 5 p.m. each day by metabolic cages. An equal volume of urine from each mouse maintained on a high salt diet during the last 5 days before Tempol treatment or the last 5 days after Tempol treatment was used to EV isolation. The urine samples were subject to centrifugation at 1000× *g* for 15 min at 4 °C. The resulting supernatant was then filtered using a 0.2 µm rapid-flow Nalgene filter (Thermo Fisher Scientific) before being subject to ultracentrifugation at 52,000 rpm for 90 min at 4 °C using a fixed-angle Ti-70 rotor (Beckman Coulter, Inc., Brea, CA, USA). The EV pellet was resuspended in 30 mL of ultra-pure 1X phosphate buffered saline (PBS) (ThermoFisher) and subject to ultracentrifugation again at 52,000 rpm for 90 min at 4 °C using the same rotor. The final EV pellet was resuspended in 200 µL of 1XPBS and frozen at −80 °C.

### 2.10. Nanoparticle Tracking Analysis

A NanoSight NS300 (Malvern Panalytical, Malvern, UK) machine loaded with NTA 3.4 Build 3.4.4 Software (Malvern Panalytical, Malvern, UK) was used to measure EV size and concentration at 25 °C. A NanoSight NS300 was equipped with a syringe pump to infuse each sample (1:1000 dilution in filtered 1XPBS (Thermo Fisher Scientific, Waltham, MA, USA) at a continuous rate of 65 while 3 separate videos were recorded. This pump infusion affords better statistical reproducibility by measuring more particle tracks within the same video length. The NTA software later processed the data to determine the overall Mean, Mode, and Standard deviation across the three videos as Merged Data. Separately, the NTA software also reported the mean and standard error across the videos as three separate sums. The number of significant digits in the standard error was not more than the minimum number of significant digits in the data set. The mean and error of 5 biological samples in each group was calculated in SigmaPlot and the size was then rounded to the nearest tenth.

### 2.11. Extraction of Lipids from Urinary EVs

EVs were extracted for lipids using the Bligh and Dyer method [15] with minor modifications, as described previously [16]. Briefly, 6 µL of the EV solutions (containing 6.21 × 10^9^ ± 1.48 × 10^9^ and 2.4 × 10^9^ ± 1.37 × 10^8^ particles for before and after Tempol treatment, respectively) were adjusted to 1 mL using water in a 10 mL glass screw-capped tube. After keeping on ice for 10 min, 2 mL methanol and 0.9 mL methylene chloride were added to the tube and vortexed for 30 s. The EquiSPLASH Lipidomix (Avanti Polar Lipids, Inc., Alabaster, AL, USA) mixture was used as an internal standard, which consists of a mix of 13 deuterated lipids, each at 100 µg/mL concentration. Deuterated lipids were d18:1-18:1(d9) sphingomyelin (SM); C15 ceramide (CER)-d7; 15:0-18:1(d7)-15:0 triacylglycerol (TAG); 15:0-18:1(d7) diacylglycerol (DAG); 18:1(d7) monoacylglycerol (MAG); 18:1(d7) cholesteryl ester (CE); 15:0-18:1(d7) phosphatidylcholine (PC); 18:1(d7) lysophosphatidylcholine (LPC); 15:0-18:1(d7) phosphatidylethanolamine (PE); 18:1(d7) lysophosphatidylethanolamine (LPE); 15:0-18:1(d7) phosphatidylglycerol (PG); 15:0-18:1(d7) phosphatidylinositol (PI) and 15:0-18:1(d7) phosphatidylserine (PS). The stock of internal standards was diluted five times and 50 µL (consisting of 20 µg/mL each standard) was added to each sample and incubated at room temperature for 30 min. Then, 1 mL water and 0.9 mL methylene chloride were added and tubes were gently inverted 10 times and centrifuged at 200× *g* for 10 min. The organic lower phase was carefully collected using a glass Pasteur pipette. The second round of extraction was performed by adding 2 mL methylene chloride, inverting the tubes, centrifugating, and collecting the lower phase. The collected organic phases from 2 rounds were pooled, dried using a gentle stream of N_2_, reconstituted in 50 µL 96% ethanol, and used for LC-MS/MS analysis. We minimized the use of plastic tips in the process of lipid extraction by using Hamilton glass syringes or glass Pasteur pipettes, especially for organic solvents.

### 2.12. Liquid Chromatography Tandem Mass Spectrometry and Scanning Parameters

Extracted lipids (5 µL) were processed and analyzed, as described previously [16]. Liquid chromatography electrospray ionization-tandem mass spectrometry (LC ESI-MS/MS) was performed using ultra-high-performance liquid chromatography (UHPLC, Shimadzu Co., Kyoto, Japan) and a QTRAP 6500 mass spectrometer (AB SCIEX, Redwood Shores, CA, USA). Lipids were separated using an XBridge Amide 3.5 μm, 4.6 × 150 mm column (Waters, Ireland) with a binary gradient of mobile phase solutions. The solutions were acetonitrile: water with the ratio of 95:5 (*v*/*v*) for A and 50:50 (*v*/*v*) for B solutions, both with 1 mM ammonium acetate (pH, 8.2). A linear gradient of solvent B was set to 6% for 6 min, increased to 25% within 4 min, reached 98% within 1 min, and finally to 100% within 2 min. The flow rate was 0.7 mL.min^−1^ during lipid separation and increased to 1.5 mL.min^−1^ for 3 min at the end of each run to flush the column using mobile phase B. To avoid carryover, isopropanol was used both as a blank to run in sample intervals and for needle washing.

A scheduled Multiple Reaction Monitoring (MRM) algorithm was set to scan more than 1100 lipid species from 19 lipid classes in 4 main categories: (1) Sphingolipids including SM, CER, hexosylceramide (HCER), dihydroceramide (DCER), and lactosylceramide (LCER); (2) Glycerolipids including TAG, DAG, and MAG; (3) CE as a sterol lipid and (4) Glycerophospholipids including PC, LPC, PE, LPE, PG, PI, PS, lysophosphatidylinositol (LPI), lysophosphatidylglycerol (LPG), and lysophosphatidylserine (LPS). The instrument was operated in both positive and negative modes with variable collision energy ranging from 25 to 60 depending on the lipid species [17]. The declustering potential of the electrospray ionization source was set to 80 and 60 for negative and positive modes, respectively. Entrance and collision cell exit potentials were set to 10 and 15, respectively, with 4.5 kV ion spray voltage and 300 °C temperature. The instrument was operated and data were acquired using Analyst software (ver. 1.6.2). Efficiencies of extraction and ionization for lipids were corrected using a surrogate internal standard in MultiQuant software (ver. 3.0.3). The ratio of lipid peak area to the surrogate internal standard was used for lipid quantification. The concentration of lipids was expressed as nM and the percentage of each lipid species related to the total lipids was calculated for normalization. Data of 5 biological replicates and 2 technical replicates (twice injection of samples) were used to compare samples with or without Tempol infusion.

### 2.13. Statistical Analysis

All data are presented as mean values ± SEM and were compared using the Student’s *t*-test in SigmaPlot software (Jandel Scientific, CA, USA). A *p* value of < 0.05 between the groups was considered statistically significant.

## 3. Results

### 3.1. Tempol Treatment Reduces Systolic Blood Pressure in Salt-Loaded Hypertensive 129Sv Mice

**Figure 1:**
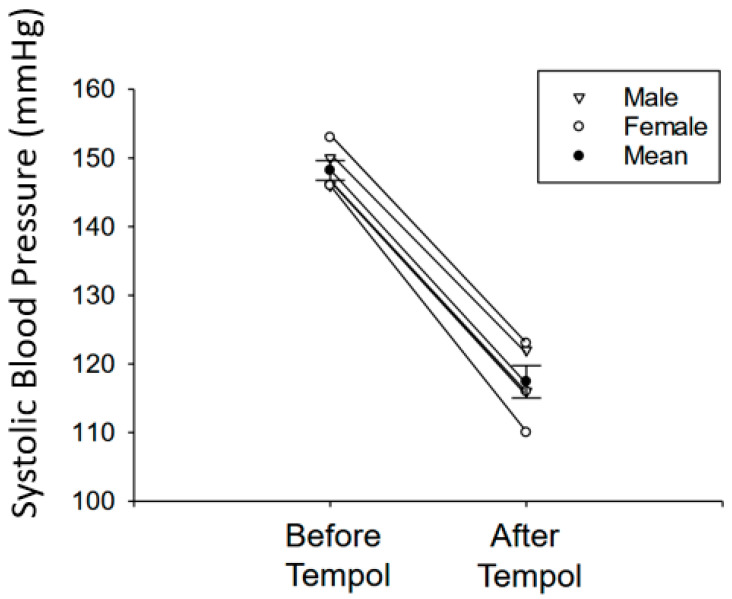
**Blood pressure of hypertensive 129Sv mice before and after Tempol infusion.** Systolic blood pressures of salt-induced hypertensive 129Sv mice before and after Tempol infusion measured by the tail-cuff method. N = 5 mice per group. A paired *t*-test was performed using the raw data. *p* = 0.063. Male mice and female mice were denoted by different symbols as indicated in the key.

Since an increase in ROS is known to contribute to the pathogenesis of salt-sensitive hypertension and renal sodium handling is directly coupled to blood pressure regulation, we further investigated whether Tempol infusion can reduce systolic blood pressure in adult 129Sv hypertensive mice after salt-loading. The basal systolic blood pressure before the 129Sv mice were salt-loaded was 120.43 ± 3.49 mmHg. Salt-loaded hypertensive 129Sv mice showed a systolic blood pressure of 148.2 ± 1.43 mmHg, but the blood pressure was reduced to 117.4 ± 2.36 mmHg after Tempol treatment (Figure 1).

### 3.2. Tempol Infusion Attenuates the Excretion of EVs in the Urine of 129Sv Mice

**Figure 2:**
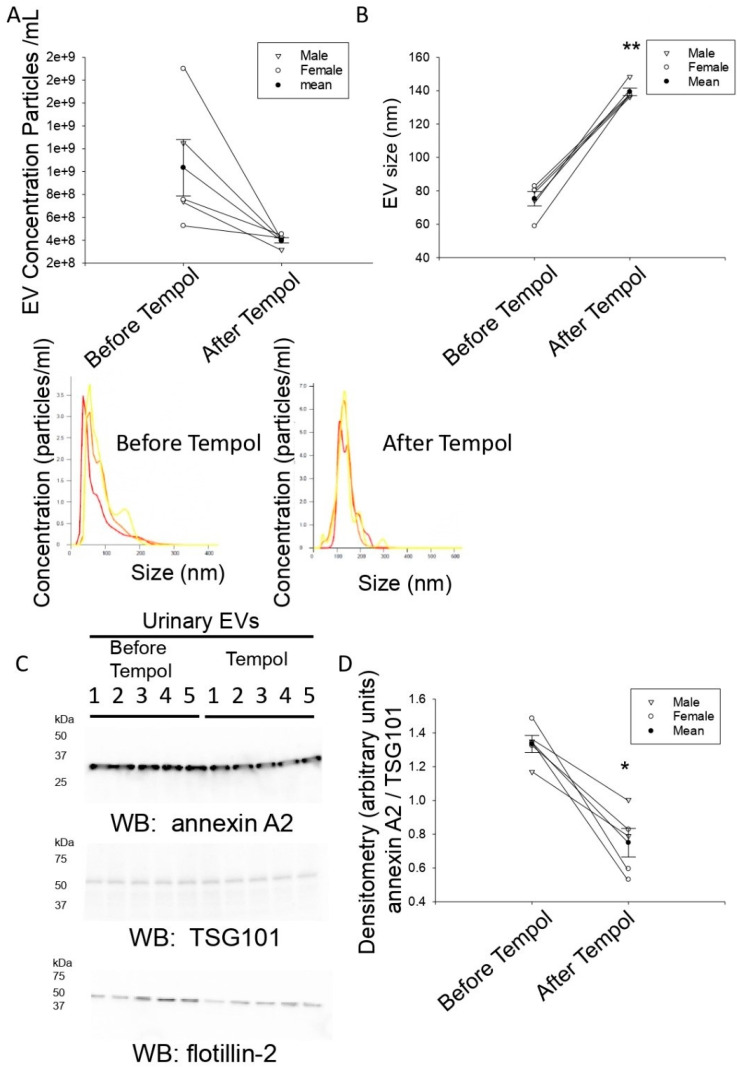
**Characterization of urinary EV concentration and size.** Urinary EVs were isolated from 24 h urine samples of salt-induced hypertensive 129Sv mice before and after Tempol infusion. Nanoparticle tracking analysis (NTA) was performed for each EV preparation in order to determine EV concentration and size. (**A**) Summary dot plot showing the concentration of urinary EV from each of the two groups. A paired *t*-test was performed and the *p*-value was 0.058. (**B**) Summary dot plot showing a difference in EV size between the two groups. A paired *t*-test was performed. Representative nanoparticle tracking images for each of the two groups is given below panel A and B. The three colors represent each sample being analyzed three times. (**C**) Western-blot analysis for Annexin A2, TSG101, and Flotillin-2 was performed to show enrichment of these EV markers in each of the EV preparations (N = 5) per group. (**D**) Densitometric analysis of the Annexin A2 immunoreactive band in panel C normalized to the TSG101 immunoreactive band. A paired *t*-test was performed. * Represents a *p*-value of < 0.05, ** represents a *p*-value of < 0.005.

Nanoparticle tracking analysis was used to measure the concentration of EVs excreted in the urine of mice before and after Tempol infusion. As shown in Figure 2A, the concentration of urinary EVs from high salt-induced hypertensive 129Sv mice decreased (4 ± 0.23 E8*particles/mL) after the mice were infused with Tempol compared to before treatment (1.03 ± 0.25 E9*particles/mL).

### 3.3. Tempol Administration Increases the Size of Urinary EVs from 129Sv Mice

We further investigated differences in EV size before and after administration of Tempol. Unlike the decrease in urinary EV concentration that was observed after Tempol administration, the size of the urinary EVs increased after Tempol treatment (139.30 ± 2.26 nm) compared to before the mice received treatment (75.26 ± 4.33 nm), (Figure 2B).

### 3.4. Reduced Annexin A2 in Urinary EVs after Tempol Treatment

In order to probe the purified EVs for the enrichment of various proteins, we lysed an aliquot of EVs in an equal volume of RIPA buffer and resolved the EV proteins by SDS-PAGE. The proteins were transferred to nitrocellulose membranes before blotting for various EV associated proteins. We attempted to investigate the enrichment of three different types of EV maker proteins. First, we chose the calcium binding protein annexin A2 because it is not only enriched in EVs, but it also plays a role in recruiting microRNAs into EVs [18]. Next, we chose the tumor susceptibility gene 101 (TSG101) protein since it is an exosomal marker that can be used for the normalization of small EV excreted into the urine [19]. Finally, we chose Flotillin-2 because it is an EV marker that is known to play a role in the composition of exosomes [20]. Western blotting for Annexin A2, TSG101, and Flotillin-2 was performed using specific validated rabbit polyclonal antibodies [21,22,23]. Although the amount of the EV TSG101 and Flotillin-2 did not change in urinary EVs isolated from urine samples before and after Tempol treatment, the amount of Annexin A2 was attenuated after Tempol treatment (0.75 ± 0.08) compared to before treatment (1.33 ± 0.05) (Figure 2C,D).

### 3.5. Quantification of EV Lipids before and after Tempol Infusion

The incorporation of EV lipids into the luminal membrane of recipient cells along the nephron and collecting duct may alter the fluidity of the lipid bilayer or the function of transporters and ion channels that contribute to sodium reabsorption mechanisms and blood pressure regulation. We used LC ESI-MS/MS to identify changes in the lipid composition of freshly isolated EVs from the urine of each animal. EV lipids were extracted and quantified in control and Tempol infused samples. Lipid species were acquired both in positive and negative scan modes from four categories of sphingolipids, glycerolipids, glycerophospholipids, and sterol lipids using targeted mass spectrometry.

**Figure 3:**
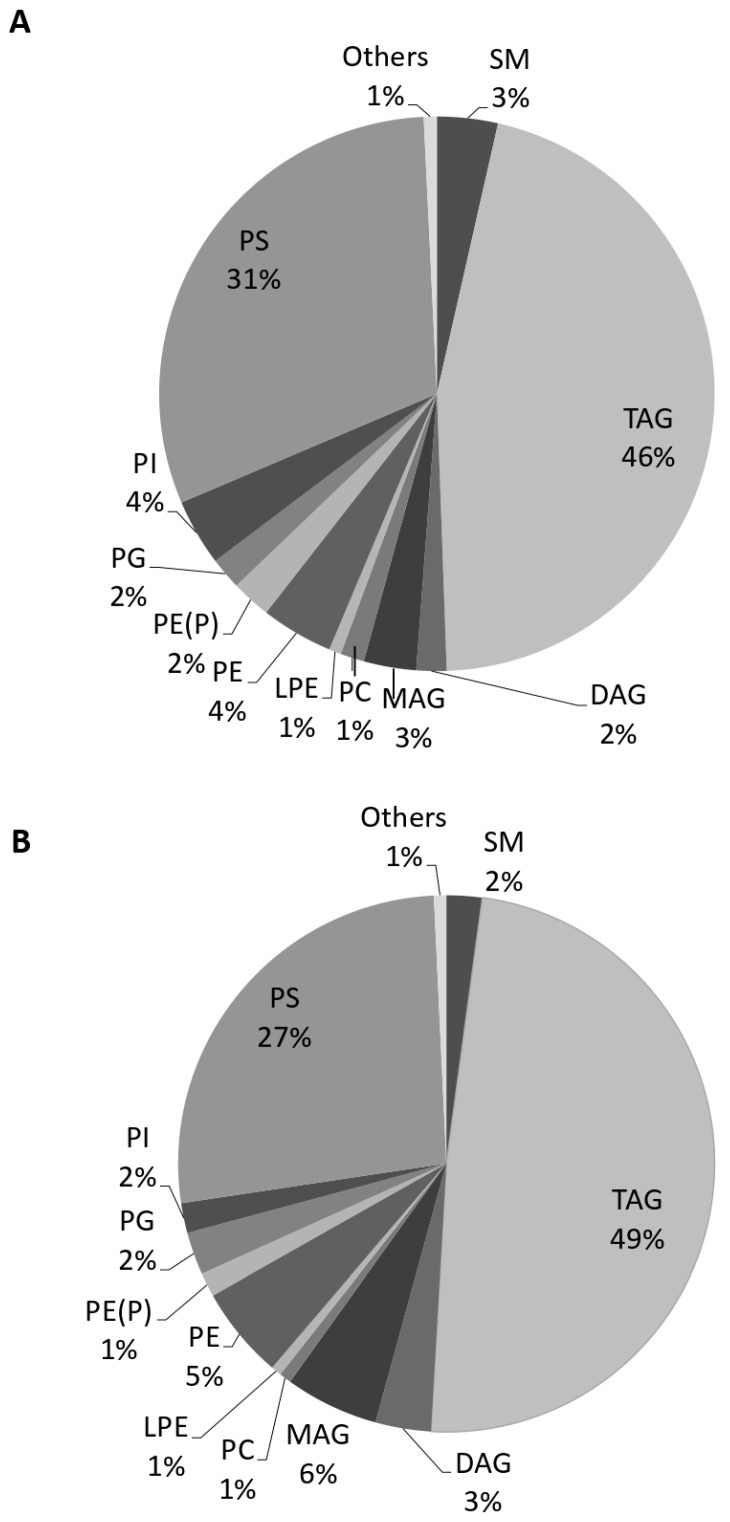
**Exosomal lipid composition with or without Tempol treatment.** Lipids were extracted from urinary EVs and quantified using targeted mass spectrometry. The total concentration of the identified lipids was calculated, and the proportion of each lipid class was presented for the samples before (**A**) and after Tempol infusion (**B**).

About 98% of the number of scanned lipids were from three main categories of glycerophospholipids, glycerolipids, and sphingolipids, according to the targeted method. The compositions of the number of identified and significantly changed lipids were similar, which were 60%, 29%, and 10% for glycerophospholipids, glycerolipids, and sphingolipids, respectively (Table 1). Lipids were quantified according to the given concentration of surrogate internal standards. The concentrations of all lipid classes were summed up and the proportion of each lipid class was calculated as a percent of total for each group. Glycerolipids and glycerophospholipids comprised 95% of the total identified lipid in the EVs (Figure 3). In this study, five phospholipids, including PC, PE, PG, PI, PS, and alkyl and alkenyl forms of PS (PE(O) and PE(P), respectively) as well as two lyso- forms (LPC, and LPE) were identified.

Tempol treatment changed the composition of EV lipids by increasing the concentrations of DAG and MAG and reducing the concentration of TAGs. Out of 202 quantified glycerophospholipids, the concentration of 22 and 65 lipid species was increased and decreased, respectively (Table 1). In glycerolipids, including TAG, DAG, and MAG, out of the 98 identified lipids, the quantities of 11 and 29 lipids were increased and reduced, respectively. SM, CER, HCER, and DCER were sphingolipids in which out of the 32 identified lipids, the concentration of 14 lipids were reduced after Tempol treatment. Two CE lipids belonging to the sterol lipids class with no changes in the concentration were detected in both groups (Table 1). Lipid species with a significant change in their concentration after Tempol infusion are presented in supplementary Table 1 (URL: https://figshare.com/s/18f329e9dd705b7741c5 (accessed on 29 November 2021); doi:10.6084/m9.figshare.13488465).

**Figure 4:**
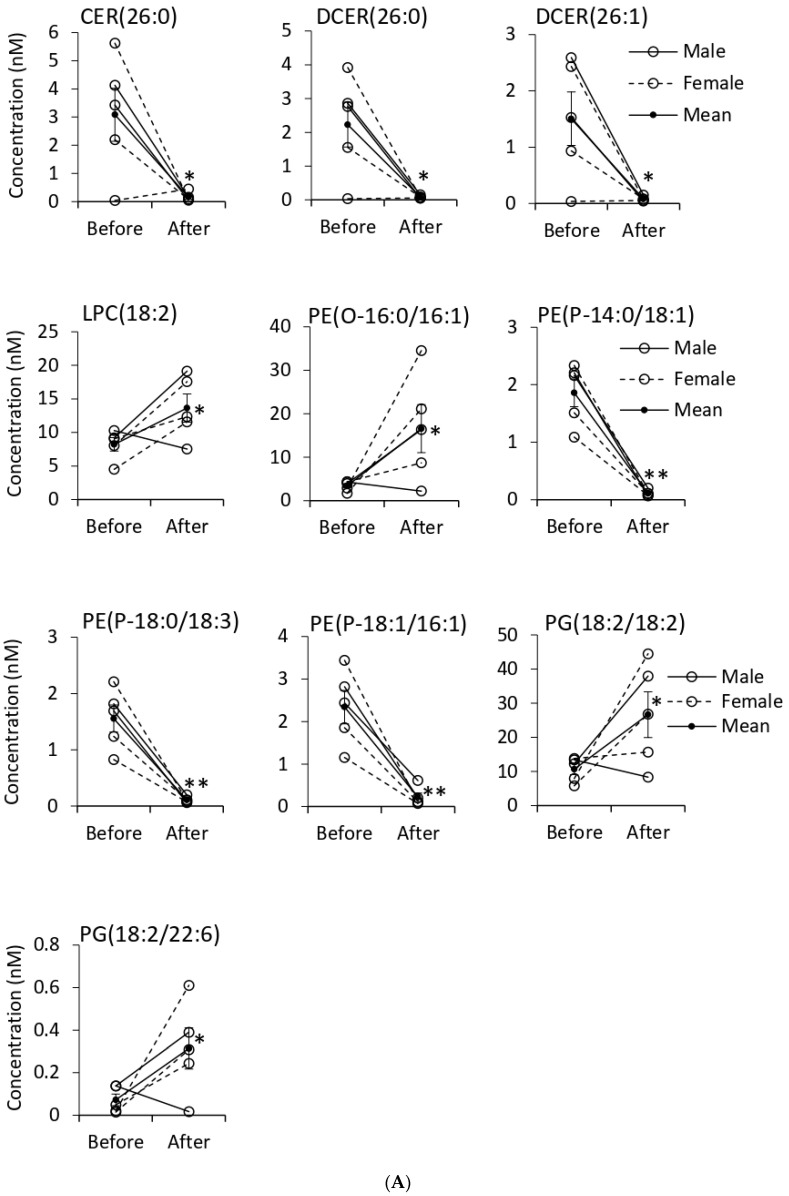

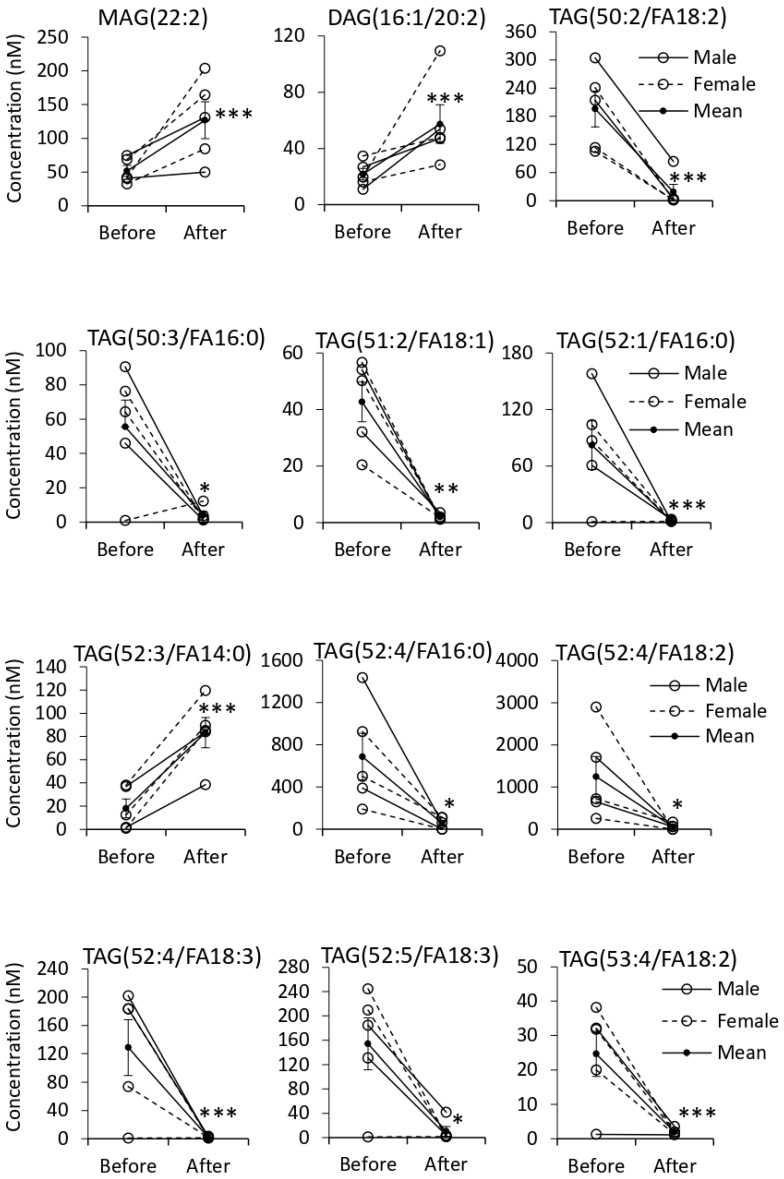

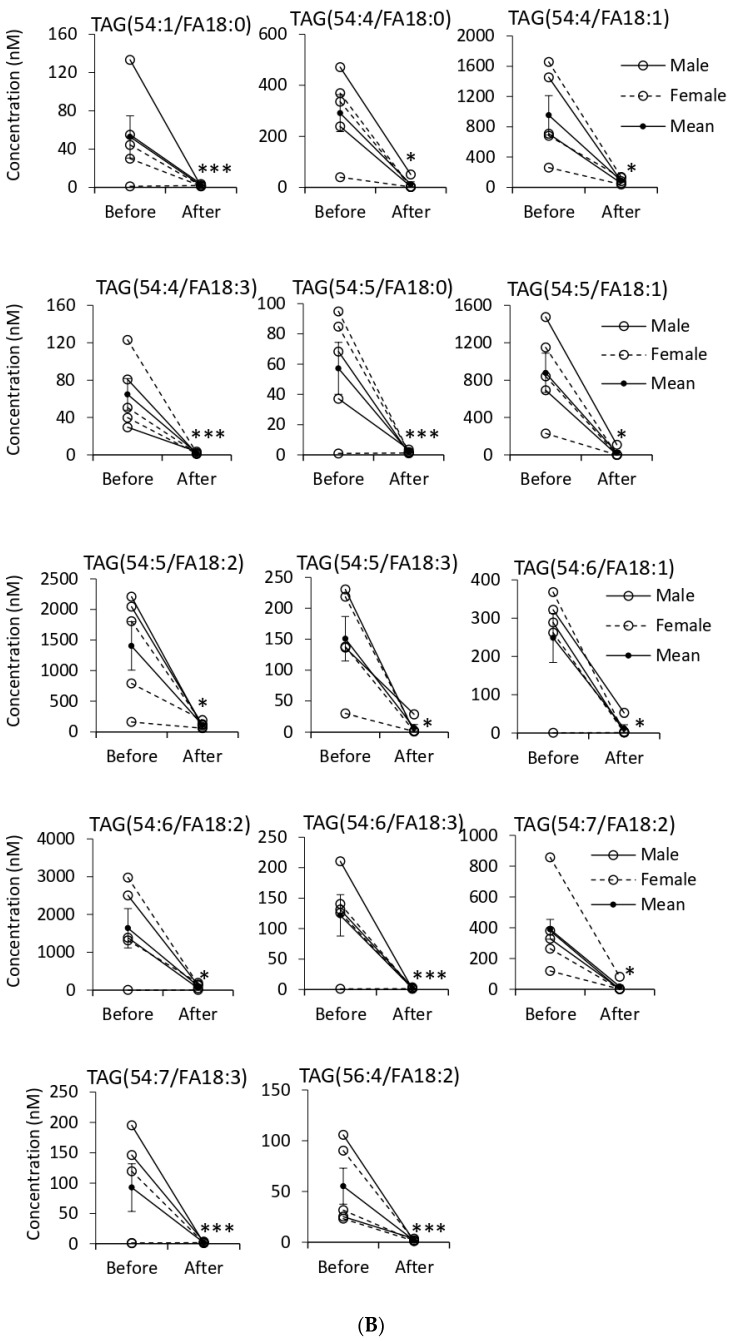
**Shortlist of the exosomal lipids affected by Tempol infusion.** Urinary EVs lipids were quantified based on the concentration of the surrogate internal standards. Sphingolipids and glycerophospholipids (**A**) and glycerolipids (**B**) with an increase in the concentration or >10X decrease in the concentration after Tempol treatment were presented. A paired *t*-test was performed to compare the two groups. * Represents a *p*-value of <0.05, ** represents a *p*-value of <0.005, *** represents a *p*-value of <0.0005.

We provide a shortlist of significantly changed lipids with more 2 two times increase or more than 10 times decrease (Figure 4). Among glycerolipids, the concentration of TAG(52:3/FA14:0), DAG(16:1/20:2) and MAG(22:2) and among glycerophospholipids, LPC(18:2), PE(O-16:0/16:1), PG(18:2/18:2), and PG(18:2/22:6) increased more than two times after Tempol infusion. On the other hand, the concentration of 23 TAG, three PE(P), one CER, and two DCER decreased more than 10 times after the treatment (Figure 4).

### 3.6. Tempol Treatment Increases Urinary Sodium Concentrations but Does Not Affect Urine Osmolality in Hypertensive 129Sv Mice

**Figure 5:**
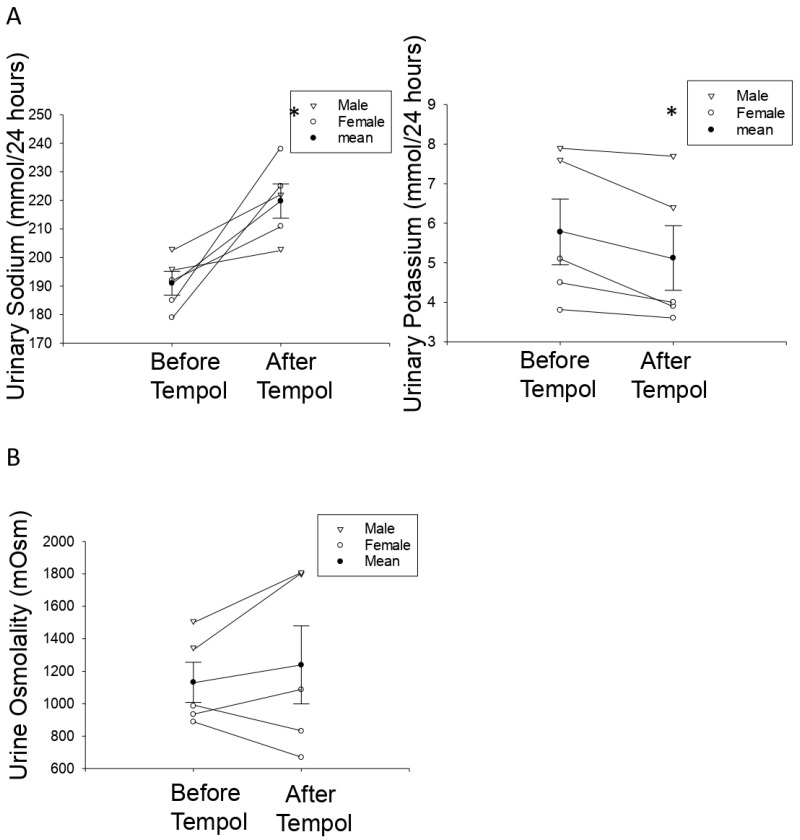
**Urinary electrolyte concentrations in hypertensive 129Sv mice before and after Tempol infusion:** (**A**) Urinary sodium concentration (**left**) and urinary potassium concentration (**right**) for salt-induced hypertensive 129Sv mice before and after Tempol infusion. (**B**) Urine osmolality for salt-induced hypertensive 129Sv mice before and after Tempol infusion. N = 5 mice per group. A paired *t*-test was performed and a * represents a *p*-value <0.05.

Water intake and urine production were measured and we observed male and female mice produced about 1.5–4 mL of urine per day while maintained on a normal salt diet. The amount of urine produced from one week after the mice were maintained on a high salt diet until the end of the study was greater than 6.5 mL per day. The effect of Tempol treatment on urinary electrolytes in salt-loaded hypertensive 129Sv mice has not been investigated. Here, we investigated whether Tempol treatment affects urinary sodium and potassium levels. Tempol infusion increased urinary sodium concentrations in salt-induced hypertensive 129Sv mice (219.8 ± 13.44) compared to before the mice received Tempol treatment (191.0 ± 9.35) (Figure 5A). However, there was no change in urine osmolality before and after treatment (Figure 5B).

### 3.7. Tempol Treatment Decreases Renal ENaC Alpha Protein Expression and Proteolysis in Hypertensive 129Sv Mice

**Figure 6:**
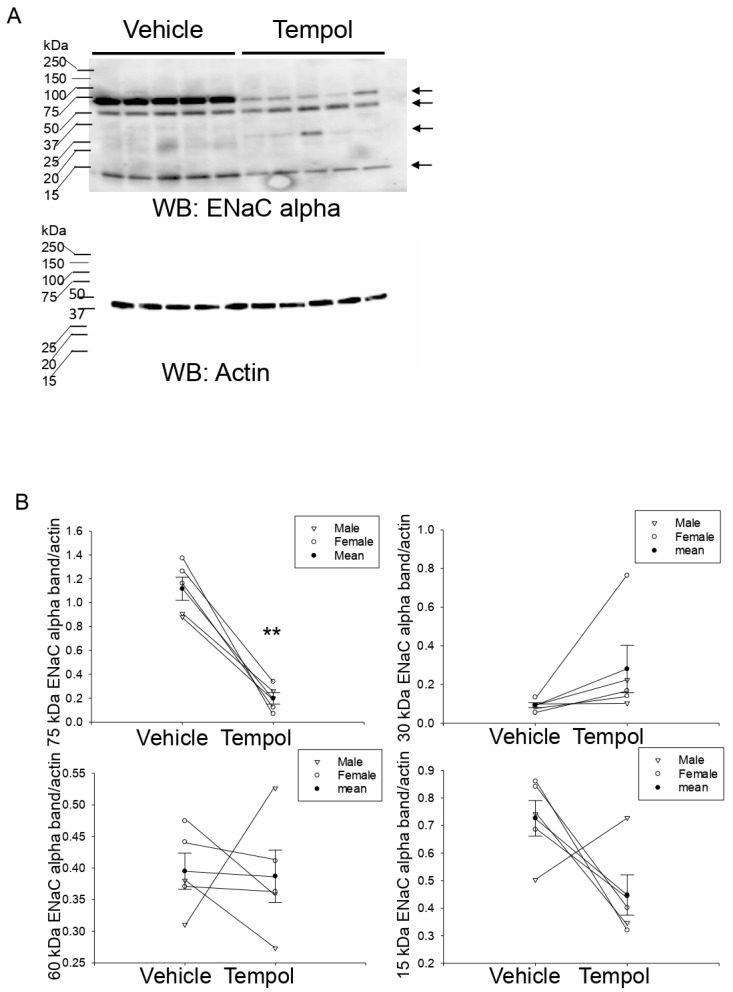
Western blot analysis of ENaC alpha protein expression in renal kidney cortex tissue of hypertensive 129Sv mice infused with vehicle or Tempol: (**A**) Western blot of ENaC alpha protein expression in kidney cortex samples from salt-induced hypertensive 129Sv mice infused with either vehicle or Tempol. (**B**) Densitometric analysis of the immunoreactive ENaC alpha band normalized to the actin band shown in panel A. N = 5 mice per group. A paired *t*-test was performed and the *p*-value was 0.063 for the 30 kDa band. ** represents a *p*-value oof <0.005.

ENaC expressed in the aldosterone-sensitive distal nephron and collecting duct plays an important role in total body sodium balance and blood pressure control. Bao et al. showed the effects of ENaC in cultured Xenopus A6 cells are abolished by Tempol treatment [24]. Zou et al. showed the ROS scavenger Tempol reverses the effect of high-salt on ENaC activity and systolic blood pressure in VDAC3-KO mice [25]. However, to our knowledge, the effect of Tempol infusion in hypertensive 129SV mice on renal ENaC protein expression and proteolysis has not been investigated. Tempol infusion in these animals resulted in a decrease of a 75 kDa and a 15 kDa proteolytically cleaved band for renal ENaC alpha compared to animals infused with vehicle (Figure 6).

### 3.8. Tempol Treatment Decreases MLP1 Protein Expression in Hypertensive 129Sv Mice

**Figure 7:**
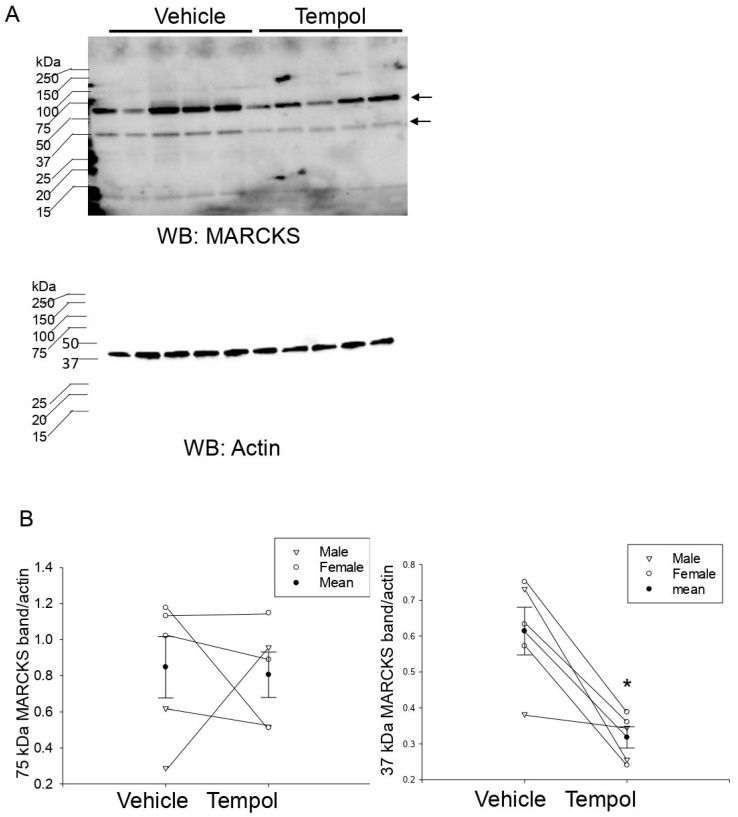
Western blot analysis of MARCKS and MLP1 protein expression in renal kidney cortex tissue of hypertensive 129Sv kidneys infused with vehicle or Tempol: (**A**) Western blot of the MARCKS family of proteins in kidney cortex samples from salt-induced hypertensive 129Sv mice infused with either vehicle or Tempol. (**B**) Densitometric analysis of the immunoreactive MARCKS (75 kDa) band and the MLP1 (37 kDa) band normalized to the actin band in panel A. N = 5 per group. A paired *t*-test was performed, and * represents a *p*-value <0.05.

In order to further investigate the mechanism by which Tempol could be regulating the density of renal ENaC alpha protein expression at the luminal membrane, we investigated the regulation of proteins that interact with ENaC and stabilize its expression at the luminal membrane. The family of MARCKS proteins expressed in the kidney includes MARCKS and MLP1. These proteins directly bind to ENaC subunits and anionic phospholipid phosphates such as PIP2 and maintain the channel in an open confirmation at the luminal membrane [8,9]. We investigated changes in MARCKS and MLP1 protein expression in kidney cortex samples of high salt-induced hypertensive 129Sv mice infused with either Tempol or vehicle using a rabbit polyclonal antibody that we validated (Supplemental Figure S1). Similar to ENaC alpha protein expression, mice infused with Tempol showed less MLP1 protein expression in the kidney cortex compared to mice infused with vehicle (Figure 7).

## 4. Discussion

EVs are nano-sized vesicles that participate in numerous physiological processes including intercellular communication, cellular differentiation, and intracellular signaling. This is the first study to investigate the regulation of EV secretion and EV lipid cargo by the ROS scavenger, Tempol in a mouse model of salt-sensitive hypertension. Here, we also investigate the ENaC dependent regulation of sodium balance and blood pressure after infusion of Tempol compared to vehicle treatment. Numerous studies have shown ROS participate in redox signaling and aberrant ROS formation and degradation has been implicated in the pathogenesis of essential hypertension. Increased ROS production leads to the conversion of NO to peroxynitrate, a toxic molecule that can modify proteins and DNA. This molecule presumably reduces the bioavailability of NO, increases vascular tone, and increases sodium retention [26].

Tempol has a protective effect on various kidney associated diseases including chronic ischemic renal injury and hypertension. Hisaki et al. showed Tempol supplementation decreases the adverse effects of salt-induced hypertension including that caused by increased oxidative stress in renal tissue, increased kidney damage, and decreased creatinine clearance in dahl salt-sensitive rats [27]. However, the effects of Tempol in salt-sensitive 129Sv mice, and on renal ENaC and proteins that regulate it, has not been investigated. Renal ENaC plays an important role in sodium balance and hence blood pressure. Our studies focused on the protein expression of the alpha subunit of ENaC, because this is the best characterized ENaC subunit, which plays a specific role in channel assembly and targeting of the channel to the luminal membrane [28].

Here, we also investigated the regulation of the MARCKS family of proteins since they play an essential role in stabilizing ENaC in an open conformation. MARCKS is an unusual protein since it’s predicted electrophoretic mobility is about 32 kDa but it migrates in SDS-PAGE gels at around 75–80 kDa, as previously shown [8,29,30]. MARCKS Like Protein 1 (MLP1), also called MacMARCKS or MARCKS related protein, has a similar topology as MARCKS [31]. Like MARCKS, MLP1 consist of a myristoylated N-terminal domain and effector domain consisting of protein kinase C phosphorylation sites and a calmodulin binding site, both of which regulate the subcellular localization of the proteins [31]. Since MacMARCKS/MLP1 shares high homology to MARCKS but runs at approximately 45 kDa [32], it is reasonable to speculate that the immunoreactive band between 37 and 50 kDa that we observed in our Western blots is either MLP1 or a cleaved form of MARCKS.

A previous study showed type 1 alveolar epithelial R3/1 cells treated with hydrogen peroxide released a greater concentration of EVs and those EVs were larger in size compared to cells treated with vehicle. This suggests EVs are formed and released in response to oxidative stress in type 1 alveolar cells. In this study, we found that the size of urinary EVs isolated from hypertensive 129Sv mice increased after Tempol administration. This would imply that the larger EVs would be enriched in various cargo including specific proteins, lipids, and miRNAs. Our Western blots of annexin A2 showed a decrease in the amount of protein within EVs after Tempol treatment. The amount of two other EV proteins, TSG101 and flotillin-2, did not change after Tempol treatment. Additional proteomic studies can be performed to further investigate changes in the number of various proteins within EVs before and after Tempol treatment.

Our results suggest that Tempol alters the lipid profile of urinary EVs from salt-induced hypertensive 129Sv mice. All three main lipid categories, sphingolipids, glycerolipids, and glycerophospholipids were affected by the treatment. A decrease in TAG content was almost at the same percentage as an increase in DAG and MAG contents after Tempol infusion, resulting in a similar concentration of total glycerolipids in both groups. TAG(52:4/FA18:3) and TAG(54:6/FA18:3) were reduced more than 40 times and 15 other TAG species were undetectable after the treatment. On the other hand, DAG and MAG increased more than 2–10 times by Tempol infusion. We assume that the storage lipid TAG was converted to the signaling molecules of DAG and MAG after the treatment. Eichmann and Lass, explained the formation of DAG and MAG from TAG in the cell [33].

CER and DCER were other bioactive lipids that were significantly reduced after Tempol treatment. These lipid classes belong to the sphingolipid family and serve as precursors for other biologically active sphingolipids, including sphingosine and sphingosine-1-phosphate [34]. In this study, all identified CER and two of DCER were significantly decreased after Tempol treatment. Three of the species, including CER(26:0), DCER(26:0), and DCER(26:1), decreased more than 10-fold. Spijkers et al. explained the correlation between hypertension and CER content and reported that CER increased in arterial tissue of hypertensive rats [35]. Interestingly, Tempol treatment decreased the blood pressure in 129Sv mice from our study and caused a dramatic reduction in CER concentrations, which supports previous studies on the link of hypertension with CER concentrations.

One limitation of this study includes not investigating whether specific bioactive lipids, such as arachidonic acid and prostaglandins, are regulated by Tempol treatment in the kidney because a different method is required for the analysis of those bioactive lipids. These lipids will be investigated in future studies. We collected urine samples, measured blood pressure, and euthanized all animals in this study at the same time of day in order to maintain rigor and reproducibility, since both ENaC and MARCKS proteins are regulated over a 24 h cycle in a circadian dependent manner. However, future studies will further investigate whether the effects of Tempol on electrolyte balance, blood pressure regulation, and the activity of ENaC and MARCKS are regulated by core proteins of the circadian clock.

Interestingly, Western-blot analysis of TSG101 and flotillin-2 did not show any appreciable differences between the vehicle and tempol treated animals. However, there was a decrease in Annexin A2 (Figure 1D). This is probably due to Annexin A2 being an exosomal protein that is regulated differently than TSG101 and flotillin-2. For example, the extracellular trafficking of Annexin A2 was shown to be dependent on phosphorylation of the protein [36] and the levels of annexin A2 in EVs were found to be correlated to the expression level in cells that release the EVs [18].

Taken together, our study showed for the first time that Tempol decreases urinary EV excretion and alters various exosomal lipid concentrations in salt-induced hypertensive 129Sv mice. This study also showed that Tempol infusion increases urinary sodium levels without altering urinary potassium excretion (data not shown) or osmolality and decreases ENaC alpha and MLP1 protein expression in the renal cortex of 129Sv hypertensive mice. The proposed mechanism for renal ENaC down-regulation by EVs enriched in DAGs that are released during Tempol infusion warrants further investigation. The significant reduction in blood pressure upon Tempol infusion reaffirms its anti-hypertensive effects as an ROS scavenger.

## Figures and Tables

**Table 1:** Number of scanned and identified lipids in mice EVs with or without Tempol infusion.

Lipids (Category)	Scan Mode	Number of Scanned Lipids	Number of Identified Lipids *	Change in Concentration after Treatment
(Glycerophospholipids)				
Phosphatidylcholine	Neg.	79	17(4)	9↓
phosphatidylethanolamine	Neg.	66	46(7)	18↓
PE(O)	Neg.	28	13(2)	5↑4↓
PE(P)	Neg.	48	46(4)	17↓
Phosphatidylglycerol	Neg.	78	44(5)	9↑ 13↓
Phosphatidylinositol	Neg.	77	6(1)	1↓
Phosphatidylserine	Neg.	78	13	1↓
Lysophosphatidylcholine	Neg.	16	7	5↑
lysophosphatidylethanolamine	Neg.	16	10	3↑ 2↓
lysophosphatidylglycerol	Neg.	16	-	-
Lysophosphatidylinositol	Neg.	16	-	-
Lysophosphatidylserine	Neg.	16	-	-
(Sphingolipids)				
Sphingomyelin	Pos.	12	12	5↓
Ceramide	Pos.	12	7	7↓
Dihydroceramide	Pos.	12	4(2)	2↓
Hexosylceramide	Pos.	12	9	-
Lactosylceramide	Pos.	12	-	-
(Glycerolipids)				
Triacylglycerol	Pos.	445	79(15)	2↑ 29↓
Diacylglycerol	Pos.	50	7	4↑
Monoacylglycerol	Pos.	17	12	5↑
(Sterol lipids)				
cholesteryl ester	Pos.	21	2	-
**Total**		**1127**	**334**	**33↑ 108↓**

* Number of uniquely identified lipids in control samples (not detected in treated samples) are presented in parenthesis. Neg., Negative; Pos., Positive; ↑ and ↓ indicates the number of lipids with increased and decreased in concentration, respectively after Tempol infusion.

## Data Availability

All original data including full Western blots are presented in the body of this manuscript.

## Supplementary Materials

The following are available online at https://www.mdpi.com/article/10.3390/biom11121804/s1, Figure S1: MARCKS antibody validation.

## Abbreviations

CE	cholesteryl ester
CER	ceramide
DAG	diacylglycerol
DCER	dihydroceramide
EDTA	ethylenediaminetetraacetic acid
ENaC	epithelial sodium channel
hAoEC	human aortic endothelial cells
HCER	hexosylceramide
LCER	lactosylceramide
LPC	lysophosphatidylcholine
LPE	lysophosphatidylethanolamine
LPG	lysophosphatidylglycerol
LPI	lysophosphatidylinositol
LPS	lysophosphatidylserine
MAG	monoacylglycerol
NO	nitric oxide
PBS	phosphate buffered saline
PC	phosphatidylcholine,
PE	phosphatidylethanolamine,
PG	phosphatidylglycerol,
PI	phosphatidylinositol
PIP2	phosphatidylinositol 4,5-bisphosphate
PS	phosphatidylserine
RIPA	radioimmunoprecipitation assay buffer
SM	sphingomyelin
TAG	triacylglycerol
