# Supplementary material for: Tempol Alters Urinary Extracellular Vesicle Lipid Content and Release While Reducing Blood Pressure during the Development of Salt-Sensitive Hypertension
Source: Biomolecules. 2021 Dec 1;11(12):1804. doi: 10.3390/biom11121804 (PMC8699083; doi:10.3390/biom11121804)
Supplement: Supplementary file 1 [file biomolecules-11-01804-s001.zip › biomolecules-1490284-supplementary.pdf]

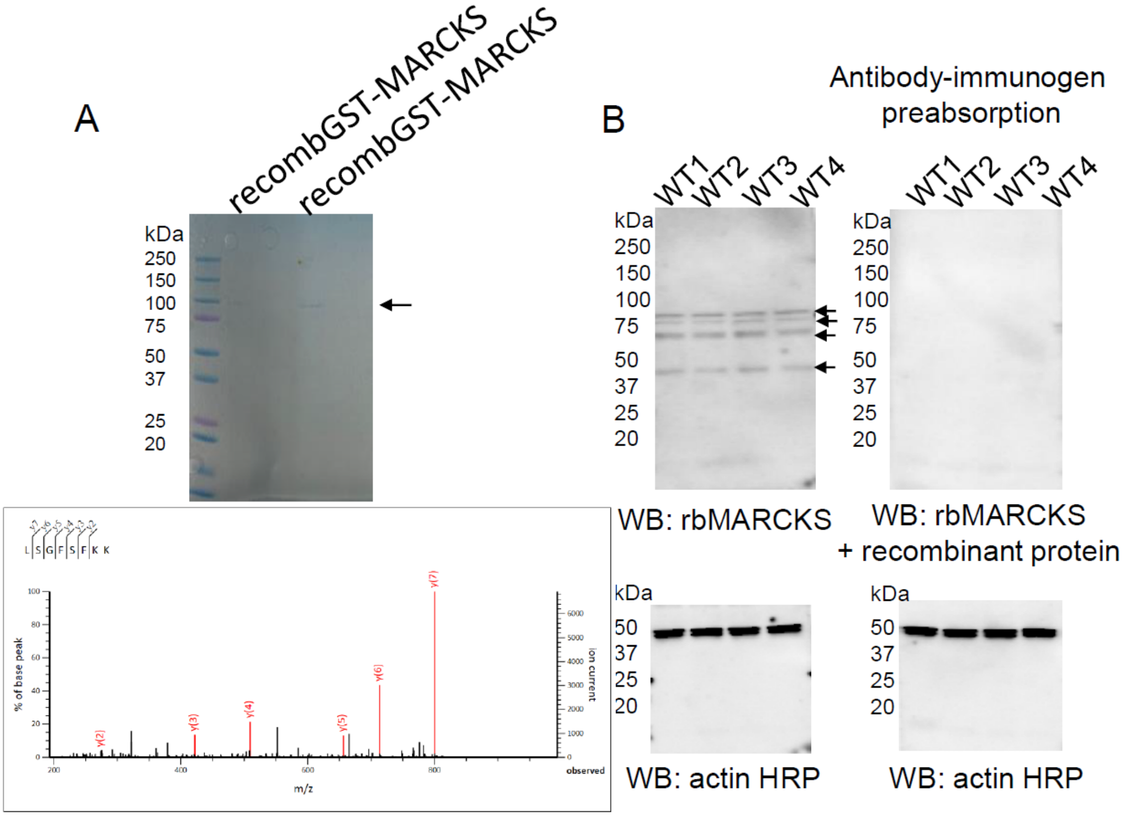

Figure S1. MARCKS antibody validation. (**A**) Mass spectrometry based detection of the Coomassie stained gel band indicated by an arrow was excised (top) and two signature peptides of MARCKS were identified (bottom). (**B**) An antibody-recombinant protein preabsorption/competition experiment was performed to demonstrate specificity of the MARCKS antibody. The antibody was pre-incubated with the recombinant protein for 4 hours at 4 °C before one blot was incubated with the pre-absorbed antibody and a similar blot was incubated with the primary antibody.
